# Challenges towards the elimination of Human African Trypanosomiasis in the sleeping sickness focus of Campo in southern Cameroon

**DOI:** 10.1186/1756-3305-7-374

**Published:** 2014-08-16

**Authors:** Gustave Simo, Jean Arthur Mbida Mbida, Vincent Ebo’o Eyenga, Tazoacha Asonganyi, Flobert Njiokou, Pascal Grébaut

**Affiliations:** Molecular Parasitology and Entomology Unit (MPEU), Department of Biochemistry, Faculty of science, University of Dschang, PO Box 67, Dschang, Cameroon; Faculty of science, University of Douala, PO Box 24157, Douala, Cameroon; National Sleeping sickness control Program of Cameroon, Yaoundé, Cameroon; Faculty of Medicine and Biomedical Science, University of Yaoundé 1, Yaoundé, Cameroon; Faculty of Science, University of Yaoundé 1, Yaoundé, Cameroon; Institut de Recherche pour le Développement, Unité Mixte de Recherche 177 IRD-CIRAD, Campus International de Baillarguet, TA A17/G, 34398 Montpellier Cedex 5, France

**Keywords:** Sleeping sickness, Socio-economic mutations, Animal reservoir, *Trypanosoma brucei gambiense*

## Abstract

The sleeping sickness focus of Campo lies along the Atlantic coast and extends along the Ntem River, which constitutes the Cameroonian and Equatorial Guinean border. It is a hypo-endemic focus with the disease prevalence varying from 0.3 to 0.86% during the last few decades. Investigations on animal reservoirs revealed a prevalence of *Trypanosoma brucei gambiense* of 0.6% in wild animals and 4.83% in domestic animals of this focus. From 2001 to 2012, about 19 931 tsetse were collected in this focus and five tsetse species including *Glossina palpalis palpalis, G. pallicera, G. nigrofusca, G. tabaniformis* and *G. caliginea* were identified. The analysis of blood meals of these flies showed that they feed on human, pig, goat, sheep, and wild animals such as antelope, duiker, wild pig, turtle and snake. The percentage of blood meals taken on these hosts varies according to sampling periods. For instance, 6.8% of blood meals from pig were reported in 2004 and 22% in 2008. This variation is subjected to considerable evolutions because the Campo HAT focus is submitted to socio-economic mutations including the reopening of a new wood company, the construction of autonomous port at "Kribi" as well as the dam at "Memve ele". These activities will bring more that 3000 inhabitants around Campo and induce the deforestation for the implementation of farmlands as well as breeding of domestic animals. Such mutations have impacts on the transmission and the epidemiology of sleeping sickness due to the modification of the fauna composition, the nutritional behavior of tsetse, the zoophilic/anthropophilic index. To achieve the elimination goal in the sleeping sickness focus of Campo, we report in this paper the current epidemiological situation of the disease, the research findings of the last decades notably on the population genetics of trypanosomes, the modifications of nutritional behavior of tsetse, the prevalence of *T. b. gambiense* in humans, domestic and wild animals. An overview on the types of mutations occurring in the region has been raised and a discussion on the strategies that can be implemented to achieve the elimination of the disease has been made.

## Introduction

Human African Trypanosomiasis (HAT), also known as sleeping sickness, is an important public health disease in sub-Saharan Africa and is responsible for a considerable degree of suffering and mortality in countries where it is endemic. On the basis of the mortality related to HAT, the disease is ranked ninth out of 25 human infectious and parasitic diseases in Africa [1, 2]. During the last decades, considerable efforts have been made in the control of this disease. The success of this control brought HAT under control and led to its inclusion in the WHO "roadmap for eradication, elimination and control of neglected tropical diseases", with a target set to eliminate HAT as a public health problem by 2020. About 8 000 new cases were reported in 2012 and the cumulative infection cases are estimated at 20 000 [3]. Presently, 36 countries have been listed by WHO as being endemic for HAT. The chronic form of HAT is reported in 24 countries and accounts for more than 90% of reported cases of sleeping sickness. Among the 24 countries affected by *Trypanosoma brucei gambiense* (*T. b. gambiense*), seven (Angola, Democratic Republic of Congo, Sudan, Chad, Central African Republic, Congo, and Uganda) contribute 98% of all reported cases of the Gambian form of sleeping sickness [2]. Thirteen countries are endemic for the Rhodesian form of sleeping sickness: most of the new cases are reported by Uganda and Tanzania. The disease cycle includes the trypanosome (*T. b. gambiense* or *T. b. rhodesiense*), the tsetse fly, and the host (human or animal). HAT occurs generally in rural areas, and during the end of the 20^th^ century, many outbreaks were observed in historical foci [4]. Faced with these outbreaks, considerable efforts have been deployed during the last two decades to fight the disease. The keystones of interventions against HAT are case detection and treatment, vector control, and management of the animal reservoir. However, in most affected countries, case detection and treatment constitutes the cornerstone of disease control. These control measures enabled a reduction of 69%, in the number of new cases reported during the period from 1997–2006 [5]. In some foci like Campo in the forest region of southern Cameroon, the disease prevalence remains relatively constant (between 0.3 to 0.86%) despite the control measures.

Currently, the elimination of HAT has been declared for 2020 and Cameroon belongs to countries targeted for this elimination. To achieve the elimination goal, a slow return of the disease over time (resurgence) in most endemic foci must be avoided through the development of new control and surveillance strategies. For this development, the epidemiology and the transmission of the disease must be well understood, especially in areas subjected to considerable socio-economic and environmental mutations like the Campo HAT focus. For instance, the urbanization, human population growth, climate change and economic development cause major environmental modifications, which have important repercussions on the epidemiology of HAT [6, 7]. In the process leading to the elimination of HAT, these environmental modifications must be taken into account, especially in foci subjected to deep mutations.

The Campo HAT focus is located in the forest region and represents the most active focus of Cameroon. During the last few decades, entomological, parasitological and genetic investigations enabled to understand the spatial and temporal distribution of tsetse, their nutritional behavior [8, 9], the animal reservoir of the disease [10–12], the population genetics of trypanosomes [10, 13–16] and the transmission cycles of the disease [8]. Putting together these data will enable us to better understand the epidemiology of the disease and also to define strategies that may lead to the elimination of HAT. However, the Campo HAT focus is subjected to socio-economic and environmental mutations, which can modify the epidemiology and the transmission of HAT at any time.

In this review, we report the current epidemiological situation of HAT at Campo, the research findings obtained during the last decades, the socio-economic and environmental mutations occurring in this region. At the end, we present strategies that could be implemented in order to achieve the elimination goal.

### Geographical localization of the Campo HAT focus

**Figure 1:**
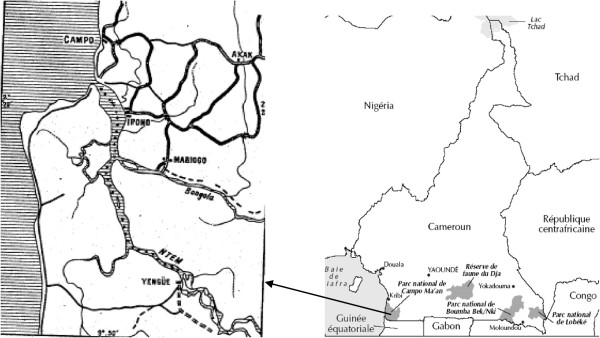
**Map showing the geographical localization of the sleeping sickness focus of Campo.**

The Campo HAT focus lies along the Atlantic coast and extends along the Ntem River, which constitutes the Cameroonian and Equatorial Guinean border. It is made up of several villages located along the coast and the roads (Figure 1). It is a hypoendemic focus where no epidemic outbreak has been reported for several years [17]. Campo is located in the equatorial rain forest characterized by a typical maritime equatorial climate with four seasons. The main activities of Campo inhabitants are fishing, picking, hunting and farming. The region of Campo has a dense hydrographic network with several rivers, swampy areas and marshes. The vegetation is a less degraded dense equatorial forest with farmlands, swampy areas and marshes along the coast. The Campo HAT focus is located near the Campo/Maan national park. The wild fauna composition is highly diversified [11, 18] and since 1932, Campo has been considered as a wild fauna reserve. Through the "Ntem" river, important population movements are observed between Campo Beach (Cameroon) and Rio Campo (Equatorial Guinea) for economic purposes. This region is subjected to considerable socio-economic and environmental mutations including the reopening of a new wood company "SCIEB", installation of the army, creation of new farmlands, construction of an autonomous port at "Kribi" and a dam at "Memve’ ele". These mutations may have impacted the epidemiology of many diseases.

## Review

### HAT cases reported in the Campo sleeping sickness focus from 1998 to 2011

The Campo HAT focus is known since the beginning of the last century and Letendu reported a prevalence of 2.9% in 1934. Between 1977 and 1997, the prevalence of the disease was between 0.2% and 0.5% [19]. In 1998 where an active case detection was undertaken, the real epidemiological situation of the disease was obtained with 16 patients detected out of about 5255 persons examined, giving a prevalence of 0.3%. Since 1998, HAT patients are reported each year (Table 1), except 2000, 2001 and 2005 where no survey was undertaken. From 1998 to 2011, about 117 HAT patients were diagnosed (Table 1); giving thus a prevalence between 0.3 to 0.86%. This indicates a permanent transmission of the disease despite active case detection and treatment. In such context, case detection and treatment cannot lead to the elimination of the disease in the Campo HAT focus. The necessity to improve control measures by developing new strategies that take into account all factors involved in the disease transmission cycle is urgent. Studies undertaken in the Campo HAT focus have generated a considerable number of parasitological, genetic and entomological data that enabled us to better understand the epidemiology of the disease. If these data are put in the current socio-economic and environmental context, the epidemiology of the disease will be well understood and control measures could be improved.

**Table 1:** **Number of sleeping sickness patients detected in the Campo HAT focus and the prevalence of the disease from 1998 to 2001**

Year of medical surveys	Number of persons examined	Number of HAT patients detected	Prevalence of the disease
1998	5255	16	0.3%
1999	No medical survey	2*	-
2000	No medical survey	-	-
2001	No medical survey	-	-
2002	1386	10	0.72%
2003	2089	15	0.71%
2004	2647	18 (4*)	0.68%
2005	No medical survey	-	-
2006	3284	7	0.21%
2007	4321	3	0.07%
2008	3528	6 (2*)	0.17%
2009	3120	16 (6*)	0.51%
2010	1742	15 (3*)	0.86%
2011	2966	9	0.3%

*: Number of patients detected through passive case detection.

### Knowledge acquired during the last decade

During the last decades, studies on the population genetics of tsetse flies and trypanosomes, analysis of tsetse flies blood meals, and investigations on the domestic and wild animal reservoirs of the disease were undertaken to improve our understanding of the epidemiology of the disease in the Campo HAT focus [8–11, 19, 20].

#### Trypanosome genetics

For population genetics studies, about 16 isolates including 13 isolates from humans, one from pig and two from crocodiles were genetically characterized using isoenzyme electrophoresis [10, 13], mobile genetic element PCR (MGE-PCR) [14], Amplified Fragments Length Polymorphism [15], microsatellite DNA markers [16], fluorescent fragment length barcoding and phylogenetic analysis of glycosomal glyceraldehyde phosphate dehydrogenase, and 16S ribosomal RNA genes [21]. The characterization of human isolates showed that they belong to *T. b. gambiense* group 1 with little genetic variability between isolates [13–16]. The characterization of crocodile isolates showed that they belong to *Trypanosoma grayi*[21] while the isoenzymes showed that the pig isolate belonged to group 1 *T. b. gambiense*[9]; indicating an animal reservoir of HAT in the Campo HAT focus. These results opened a framework for investigations on animal reservoir of HAT in this focus. In this light, molecular tools were used to search for *T. b. gambiense* in domestic and wild animals [11, 12, 22, 23].

#### Investigations on animal reservoirs of *T. b. gambiense*

For these investigations, about 464 animals including 310 domestic animals and 154 wild animals were sampled [11, 12, 22]. The use of molecular tools enabled us to report a prevalence of *T. b. gambiense* to 0.6% in wild animals and 4.83% in domestic animals; showing thus the presence of domestic and wild animal reservoirs of *T. b. gambiense* in the Campo HAT focus. If *T. b. gambiense* is able to pass from animals to humans or from humans to animals, its transmissibility index will be increased as reported by "Van Hoof [24]". If such events occur, many more cases would have been expected. However, the epidemiological situations that are observed in most HAT foci seem to show that this phenomenon does not occur regularly. The reasons explaining why *T. b. gambiense* cannot be easily transferred from animal to human is not well understood. Nevertheless, it has been shown that *T. b. gambiense* group 1 stocks isolated in pig could remain infective because it has been demonstrated that this trypanosome conserves its infectivity to humans even after 10 cyclical transmissions in pigs [25]. In such context, *T. b. gambiense* can pass from animals to humans. Such events can occur if interactions exist between human and animal transmission cycles. For instance, the socio-economic and environmental mutations that occur in the Campo HAT focus may play in favor of such interactions. In such context, the transmission may become more important because tsetse have the possibility to become infected from human and different animal species. If such events occur, a flare up of the disease can be observed because the infectivity of *T. b. gambiense* will be enhanced by its passage between man, tsetse and domestic or wild animals.

#### Entomological surveys

From 2001 to 2012, no vector control was undertaken in the Campo HAT focus although 12 entomological surveys were undertaken for research purposes [8, 9, 20, 26]; Simo, unpublished data; Grebaut, unpublished data. During these surveys, about 19 931 tsetse flies were collected (Table 2) and five tsetse fly species including *G. palpalis palpalis (G. p. palpalis, G. pallicera, G. nigrofusca, G. tabaniformis* and *G. caliginea* were identified (Table 2). *G. p. palpalis*, the main vector of HAT in this focus, was the predominant tsetse species captured in this region whatever the sampling period. Looking at the percentage of tsetse species according to sampling periods, the proportion of *G. p. palpalis* increased whereas the proportion of other flies decreased from 2001 to 2012. These differences may result from the environmental modifications (deforestation and creation of farmlands) observed in this focus. Indeed, *G. pallicera, G. nigrofusca* and *G. caliginea* are zoophiles, which are more demanding of stringent ecological conditions. The impact of humans on the environment has led to the disappearance of these flies in some biotopes. With the deforestation and the creation of farmlands and animal breeding in the Campo HAT focus, the environment became more anthropised and more appropriate for the installation of *G. p. palpalis,* which can be encountered in various types of biotopes. Furthermore, since socio-economic and environmental mutations continue to occur at Campo, there is a need to follow up these mutations because they have impacts on the transmission of HAT, and can impede the achievement of the elimination goal.

**Table 2:** **Tsetse flies species collected in the Campo sleeping sickness during the last decade**

Sampling periods	*G. p. palpalis* (%)	*G. pallicera* (%)	*G. caliginea* (%)	*G. nigrofusca* (%)	*G. tabaniformis* (%)	Total
2001 and 2002: [24]	5337 (57)	3011(32)	413 (4)	670 (7)	2	9431
2002 and 2004: [7]	1641 (67)	607 (25)	93 (4)	102 (4)	-	2443
2008: [8]	3318 (94)	152 (4)	57 (2)	14 (0.4)	-	3541
2009 to 2011**	3510 (99)	12 (0.3)	9 (0.2)	7 (0.2)	3 (0.08)	3541
2012***	942 (97)	28 (2.3)	1 (0.1)	1 (0.1)	3 (0.3)	975
Total	14748 (74)	3810 (19)	573 (3)	794 (4)	6 (0.03)	19931

*G.: Glossina*; *G. p.: Glossina palpalis*; **: [Simo, unpublished data]; ***: [Grebaut, unpublished data].

#### Blood meal analysis

The analysis of blood meals provides detailed knowledge of the feeding behavior of tsetse under natural conditions and information on the disease transmission cycles. Moreover, knowledge on the feeding behavior of tsetse is considered as a prerequisite for a successful tsetse and trypanosomiasis control program. Since the behavior of tsetse for certain hosts differs according to sampling area [27], specific identification of tsetse blood meals can enable the localization of the transmission sites, understanding of HAT transmission cycles and pathogen life cycles [28]. Data generated from the analysis of blood meals can indicate the significance of animal reservoirs of HAT, and provide entomologic factors like anthropophilic index that contribute to the disease transmission [29, 30].

The two main studies undertaken on the identification of blood meal origin of tsetse in the Campo HAT focus [8, 9] have shown that tsetse feeds on humans, domestic animals such as pig, goat, sheep, and wild animals such as antelope (*Tragelaphus spekeii*), duiker (*Cephalophus dorsalis, Cephalophus silvicultor* and *Cephalophus ogilbyi*), wild pig (*Suus scrofa*), turtle (*Kinixys homeana*), and snake (*Python sebae*) [8, 9]. Despite this variety of tsetse hosts, differences were observed between the proportions of blood meals taken on these hosts. In addition, the nutritional behavior of tsetse can vary according to sampling periods. For instance, only 6.8% pig blood meals were reported in tsetse captured at Campo in 2004 [8] while there were 22% in 2008 [9]. Interestingly, up to 18.2% *G. p. palpalis* blood meals from the Campo HAT focus were from antelope (Sitatunga) in 2004 [8], whereas no blood meal from Sitatunga was identified in 2008 [9]. These differences may result from the fauna composition, human density, season, and behavior of animals. The environmental changes also have a real impact on the nutritional behavior of tsetse because the creation of farmlands induces deforestations and modifications of tsetse habitats which have direct impacts on the fauna composition. One important factor that enables us to understand environmental mutations on the nutritional behavior of tsetse is the host diversity index which is determined through the zoophilic/anthropophilic index (Za) which is defined as the ratio between the percentages of blood meals from animals to those taken on man [31]. This index has high epidemiological importance because a positive correlation was reported between Za index and the prevalence of HAT in West Africa [31]. Understanding the socio-economic and environmental mutations that occur in each region appears important for the development of new strategies that will lead to the elimination of the disease.

### Deep mutations in the Campo sleeping sickness focus and consequences on the epidemiology of the disease

The region of Campo is currently subjected to socio-economic and environmental mutations linked to the intensification of trade between Campo beach (Cameroon) and Rio Campo (Equatorial Guinea), the reopening of new wood company (SCIEB) at Ipono, the construction of an autonomous port at "Kribi" and a dam at "Memve ele". The Campo HAT focus contains several villages and the number of inhabitant varies between villages. The high concentration of the population was previously at Campo and Ipono due to the administrative position of Campo and the wood company that created considerable economic activities at Ipono. Between 2003 and 2008, this company stopped their activities and more than 70% of inhabitants left due to the fact that about 80% of inhabitants were directly (worker of the company) or indirectly (family members of the workers, business men) linked to this company. Recently, a new Company has resumed activities at Ipono and about 200 employees started activities in this village. To the 200 employees, about 300 family members (children, wife, and relatives) will be installed at Ipono. With these socio-economic mutations, activities have resumed at Ipono and stores have reopened. In addition, other activities, such as the construction of an autonomous port at "Kribi" (at around 40 kilometers in the North) and a dam at "Memve ele" (60 kilometers in the North-east), are being carried out around the Campo HAT focus. The construction of a dam and autonomous port will bring, respectively, about 1000 and 3000 employees with their families around Campo (Grebaut, unpublished data). To satisfy the nutritional needs of these populations, new farmlands have been created around Campo. In addition, to protect wild fauna, the World Wild Fauna (WWF) sustains the development of small animal breeding by encouraging local inhabitants to implement the breeding of domestic animals such as pig, goat and sheep. All these activities have real impacts on the transmission of HAT because the creation of farmlands through deforestation and the development of domestic animal breeding through WWF initiative will induce modifications not only on tsetse habitat, but also the tsetse behavior as well as the transmission of the disease. In such context, the nutritional preference of tsetse will be more diversified and the Za index will increase, as was observed from 2004 to 2008. Za index becomes high when the environment and availability of hosts (other than human) change due to the interference of human on its milieu. Since the Campo HAT focus is continuously subjected to socio-economic and environmental mutations, it is obvious that the Za will increase with time. In fact, when the host preference of tsetse is diversified, alternating in some proportions humans, domestic and wild animals, tsetse can transmit trypanosomes to man and may also maintain the animal reservoir [8]. In this case, the transmission becomes more important because the transmissibility index of *T. b. gambiense* increases when this parasite passes from animal to man or from man to animal [24]. Consequently, the number of infected patients will increase; the transmission cycles will be accelerated and more new cases and more infected and infectious animals will be observed. The disease can flare up when the prevalence of *T. b. gambiense* reaches a critical point in humans and animals. This "dramatic scenario" can happen only if interactions exist between human and animal transmission cycles. However, in the case where no interaction exists between human transmission cycle and others, an "alternative scenario" could occur in the disease focus and consequently, the massive destruction of tsetse favorable habitats due to environmental mutations such as the human pressure and the climate change could lead to auto-extinction of the disease. Such an event has already been observed elsewhere such as in Burkina Faso for instance [32]. However, it is important to point out that this auto-extinction has been observed mainly in the savannah zone where the disease shifted from the savannah areas to the forest and mangrove areas of West Africa [33, 34]. This persistence of the disease in the forest and mangrove areas of West (Guinea, Ivory Coast) and Central (Cameroon, Gabon) Africa highlights the difficulties that will be encountered in the process leading to the elimination of the disease in these particular ecosystems.

### New strategies to achieve the elimination goal

To achieve the elimination goal in the Campo HAT focus, there is need to redefine control and surveillance strategies by involving all actors that interfere with the disease transmission cycles. The considerable number of data generated during the last decades enabled us to understand the epidemiology of HAT notably the nutritional behavior of tsetse, the population genetics of trypanosomes, the animal reservoir of HAT and the disease transmission cycles. From these data, control measures could be developed to achieve the elimination goal. However, it is important to keep in mind that several socio-economic and environmental mutations occur in this region. Therefore, the strategies that may lead to the elimination process must take into account these mutations. Although the action of mobile teams must be sustained, there is a need to improve the medical surveys. For instance, before the progressive switch from active to passive medical surveys as recommended by WHO in low prevalence settings, it is important to have a good cover rate during medical surveys organized by the mobile teams. This is very important in a sleeping sickness focus subjected to deep mutations like the Campo focus where the number of inhabitants varies continuously with the socio-economic mutations (reopening of company, construction of autonomous port at "Kribi") observed in this region. In such context, the medical surveys team must re-sensitize and remake a census of the population in order to improve the coverage rate. Moreover, with the development of animal breeding, the concept of "one health" must be put in place by putting together people involved in animal and human trypanosomiasis, as well as those of WWF. Greater effort must be made for vector control, which appears as a critical step, especially in this focus, which is neighbor to a national park, where animal reservoirs of HAT have been identified and where the value of Za can increase with environmental and socio-economic mutations. The tools developed so far can enable us to evaluate the circulation of the parasite in tsetse and vertebrates and determine with precision the origin of tsetse blood meals. Associating these elements with other entomological factors such as the tsetse density and the teneral flies into a geographic information system (GIS) will enable us to identify, localize and characterize biotopes and zones of high transmission risk where vector control can be implemented. By manipulating environmental and biological data into a GIS, it will become possible to improve control measures by reducing vector control costs if this control is focused on target zones. Using these data in a modeling system will be also very useful for predicting the evolution of the disease and the efficiency of the control measures. The use of this approach may enable us to achieve the elimination goal and to avoid the reemergence of the disease in a particular region.

## Conclusion

This review has described the current epidemiological environment within and around the sleeping sickness focus of Campo. It highlighted that considerable efforts must be put in place to understand the epidemiological impacts of deep mutations that occur in this area. It highlighted also that a better understanding of these mutations in the concept of "one health" is important for the development of appropriate strategies that will lead to achieve the elimination goal of HAT in this particular focus.
